# Computer-Aided Molecular Design Meets Network Toxicology and Molecular Docking: A Joint Strategy to Explore Common Molecular Mechanisms of Phthalates on Human Breast Cancer and Structure–Activity Relationship

**DOI:** 10.3390/ijms26209878

**Published:** 2025-10-10

**Authors:** Xinyu Yang, Zijun Bai, Xiaoyun Yan, Yu Zhou, Caiyun Zhong, Jieshu Wu

**Affiliations:** 1 Key Lab of Modern Toxicology of Ministry of Education, Center for Global Health, School of Public Health, Nanjing Medical University, Nanjing 211166, China; njmu_yangxinyu@163.com (X.Y.); 19861125515@163.com (Z.B.); yanxiaoyun0424@163.com (X.Y.); zyzhouyu123@163.com (Y.Z.); cyzhong@njmu.edu.cn (C.Z.); 2 Department of Stomatology, School of Stomatology, Nanjing Medical University, Nanjing 211166, China

**Keywords:** phthalates, breast cancer, common mechanisms, network toxicology, molecular docking, computer-aided molecular design, structure–activity relationship

## Abstract

Distinct PAEs are implicated in breast cancer progression through multiple molecular pathways. This study aims to elucidate the potential mechanisms in common by which PAEs promote breast cancer progression. Dibutyl phthalate (DBP), benzyl butyl phthalate (BBP), and diethylhexyl phthalate (DEHP) were selected as representative PAE compounds. Network toxicology guided the construction of a regulatory network centered on five key transcription factor-associated genes: *TP53*, *CTNNB1*, *PPARA*, *ESR1*, and *CDKN2A*. Differential expression and survival analyses confirmed the significant impact of these hub genes on breast cancer (*p* < 0.05). Molecular docking results revealed direct interactions between the three PAEs and hub targets, while BBP had the strongest PAE-hub gene interaction and DEHP had the weakest one. Computer-aided molecular design (CAMD), combined with molecular docking, found the importance of alkyl chains and phenyl in PAE-hub gene interaction. A group addition/subtraction controlled experiment revealed that the binding affinities of modified BBP variants to hub genes are all weaker than the unmodified parent. The drop was significant whether the C17 alkyl chain was lengthened to match DEHP (*p* = 0.026) or the phenyl group was removed (*p* = 0.022). The findings provide novel insights into the mechanism in common of PAE-promoting breast cancer and offer a foundation for the unified intervention strategies and the design of safer plasticizer alternatives.

## 1. Introduction

Phthalate esters (PAEs), which are common plasticizers with an annual global consumption exceeding five million tons, are extensively used in various materials such as polyvinyl chloride (PVC) [1]. They are widely distributed in the environment due to their lipophilicity and potential for bioaccumulation, thereby creating multi-pathway human exposure routes [2]. Substantial evidence indicates that PAEs, specifically dibutyl phthalate (DBP), butylbenzyl phthalate (BBP), and diethylhexyl phthalate (DEHP), adversely affect human health, including birth outcomes, children’s development, thyroid hormone levels, endometriosis, cancer, etc. [3,4,5]. Despite current regulatory restrictions on the use of these PAEs [6,7,8], DEHP, DBP, and BBP remain prevalent environmental contaminants [9,10,11,12,13,14]. And research has shown that 10^−9^ M PAEs (BBP, DBP, and DEHP) promote tumor sphere formation, and 10^−10^ M Mono-(2-ethylhexyl) phthalate increased the proliferation of breast cancer cells, suggesting the potential harm of even low concentrations of PAEs to the human body [15,16]. Therefore, it is necessary to conduct in-depth research on the molecular mechanisms of the effects of PAEs in humans.

Breast cancer is the most common cancer among women and the leading cause of cancer death [17,18]. Epidemiological studies have demonstrated a significant association between PAEs and breast cancer [19]. However, the mechanisms through which PAEs promote breast carcinogenesis and progression remain unrevealed thoroughly. Early studies have demonstrated that PAEs, as prototypical environmental endocrine-disrupting chemicals (EDCs), can contribute to breast carcinogenesis by simulating estrogens to bind to estrogen receptors (ER) [20] and then regulating the estrogen-responsive gene expression [21]. Subsequently, accumulating evidence reveals that PAEs also facilitate breast cancer progression through molecular mechanisms independent of classical ER-mediated pathways, such as promoting the onset of breast cancer tumors via the AhR/HDAC6/c-Myc signaling pathway [22], enhancing the expansion of breast cancer stem cells through the SPHK1/S1P/S1PR3 signaling transduction pathway [23], or promoting the progression of breast cancer by inducing the expression of lymphoid enhancer-binding factor [24]. It is evident that the promotion of breast cancer by PAEs involves a complex multi-molecular and multi-pathway regulatory network. Therefore, conducting in-depth analyses of the characteristics and patterns through which PAEs promote breast cancer development, while exploring potential common targets and mechanisms of action, is crucial for developing effective prevention and control strategies against PAE exposure.

The emergence of innovative toxicological methodologies and technologies has enabled systematic investigations into common mechanisms of toxicants. Network toxicology integrates multiple signaling pathways within regulatory networks, serving as a powerful tool to systematically elucidate the signaling mechanisms of toxicants. Computational toxicology studies interactions between pollutants and cellular targets through computer simulations. Currently, molecular docking is widely used in the screening and study of endocrine disruptors, offering a partial substitute for laborious, cumbersome, and expensive experiments [25]. Computer-aided molecular design (CAMD) is commonly used in novel drug development to identify pharmacophores (i.e., molecular modification sites). This technique has now been applied to investigate the toxic mechanisms of PAEs and design novel PAE derivatives [26,27,28], establishing a new paradigm for mechanistic toxicology research on small molecules.

This study aims to identify PAEs (BBP, DBP, DEHP)-breast cancer common targets via network toxicology, characterize PAE-target interactions through molecular docking, and determine critical chemical groups influencing these interactions using CAMD to elucidate potential common mechanisms in PAE-driven breast cancer progression and the structure-activity relationship (SAR) in it.

## 2. Results

### 2.1. Common Target Genes of Three PAEs

**Figure 1:**
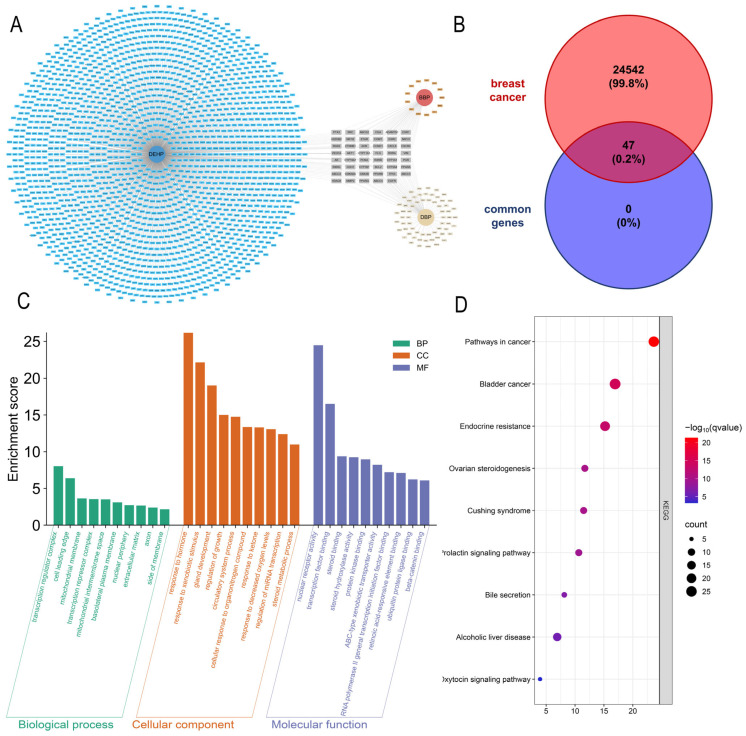
The representation of PAE exposure-gene associations (**A**) and intersection analysis of breast cancer-related genes with PAE targets (**B**). Go analysis of common genes (**C**). The *y*-axis indicates enrichment score (−log10-transformed *p*-value). KEGG analysis of differentially expressed genes (**D**). The *x*-axis indicates enrichment significance (−log10-transformed *p*-value), with color gradients reflecting adjusted significance (−log10-transformed q-value). Circle size corresponds to gene count per enriched term.

Figure 1A displays a diagram illustrating the relationship between phthalate ester (PAE) exposure and target genes. Blue boxes denote DEHP and its targets; red boxes, BBP; and yellow boxes, DBP. After consolidating and eliminating duplicate entries, a total of 2269 target genes were identified. Specifically, DBP was associated with 156 genes, BBP with 86 genes, and DEHP with 2027 genes. Centrally aligned gray boxes represent common targets of all three. The relationship diagram shows 47 shared common target genes among all three PAEs. Then these common targets were intersected with 24,589 breast cancer-related target genes, resulting in 47 potential gene targets for PAEs associated with breast cancer. (Figure 1B).

### 2.2. Functional Enrichment Analysis

Figure 1C,D displays the results of Functional Enrichment Analysis (FEA). We first examined the Gene Ontology (GO) terms enriched in the Biological Processes (BP), Molecular Functions (MF), Cellular Components (CC), and pathways in response to exposure to DBP, BBP, and DEHP. The top ten enriched BP terms included “response to hormone” and “response to xenobiotic stimulus.” The top ten CC terms included “transcription regulator complex,” “cell leading edge,” “mitochondrial membrane,” and “transcription repressor complex.” In terms of MF, “nuclear receptor activity” and “transcription factor binding” were significantly enriched. The top ten KEGG pathways included “Chemical carcinogenesis—receptor activation,” “Pathways in cancer,” “Bladder cancer,” “Endocrine resistance,” and others.

Due to the significant enrichment of transcription factors across various components, we identified 19 genes (*TP53*, *CTNNB1*, *ESR1*, *CDKN2A*, *PPARA*, *EGFR*, *AR*, *SRC*, *RXRA*, *PGR*, *PPARG*, *GSK3B*, *NR1I2*, *AHR*, *BCL2*, *PPARD*, *HDAC6*, *PCNA*, *VIM*) that were enriched in transcription factor-related components and selected them for further investigation.

### 2.3. Protein–Protein Interaction Network (PPIN)

**Figure 2:**
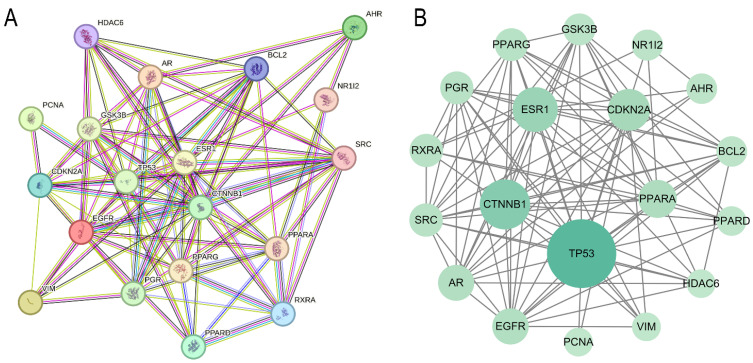
Protein–Protein Interaction (PPI) network and functional associations (**A**). Global PPI network topology with core hub genes identified through centrality analysis (inner circle) (**B**).

Using the STRING 12.0, we constructed a Protein–Protein Interaction Network (PPIN) for the 19 genes enriched in transcription factor-related components to identify the hub genes within this network. The PPIN analysis revealed 19 nodes and 98 edges, demonstrating a significant protein–protein interaction enrichment score (*p* = 3.34 × 10^−8^; Figure 2A), indicating strong interactions among all 19 genes.

Using Cytoscape 3.10.2, we predicted and visualized the hub genes within the PPIN. As shown in Figure 2B, the nodes (representing genes) are arranged in concentric circles based on their betweenness centrality rank. The color intensity and size of the nodes reflect their betweenness centrality values, with more intense and larger nodes indicating higher centrality. The top 5 genes in the inner circle, ranked by betweenness centrality, were designated as hub genes (*TP53*, *CTNNB1*, *PPARA*, *ESR1*, *CDKN2A*).

### 2.4. Differential Expression Analysis and Survival Analysis

**Figure 3:**
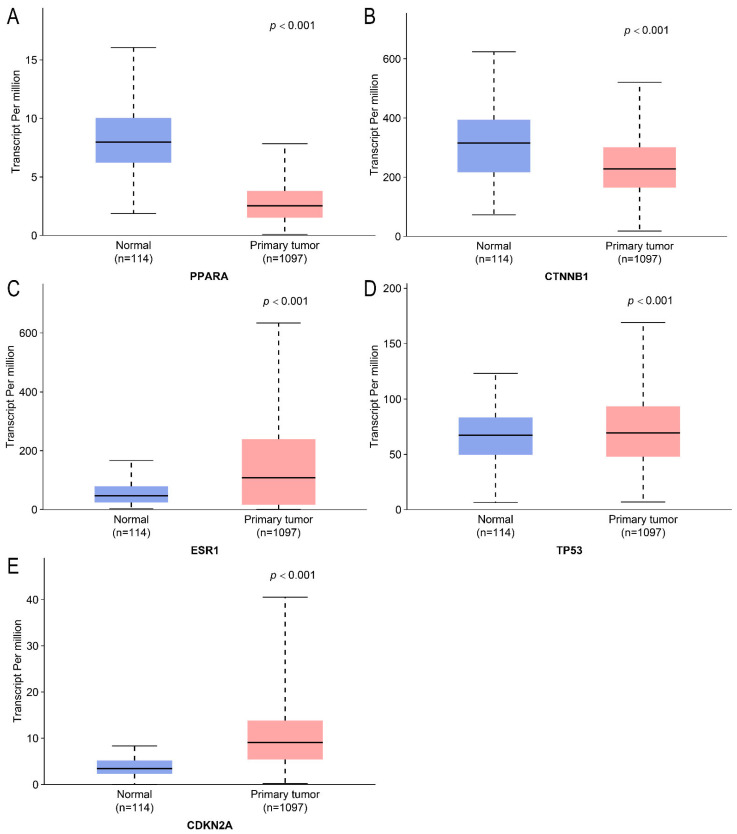
Comparative expression profiles of hub genes in tumor versus normal tissues. (**A**–**E**) correspond to *PPARA*, *CTNNB1*, *ESR1*, *TP53*, and *CDKN2A*, respectively. Differential expression levels are displayed as log_2_ (fold change) with error bars representing SEM (*p* < 0.05, *p* < 0.01).

The analysis of data from the TCGA database demonstrated significantly different expression of these genes in breast cancer tissues compared to normal tissues. Specifically, *PPARA* (*p* = 1.6524 × 10^−12^) and *CTNNB1* (*p* = 1.44033.34 × 10^−7^) were downregulated, while *ESR1* (*p* < 1 × 10^−12^), *TP53* (*p* = 6.1951 × 10^−4^), and *CDKN2A* (*p* = 1.6524 × 10^−12^) were upregulated (Figure 3). These findings illustrate an important role for these genes in tumor initiation and progression.

**Figure 4:**
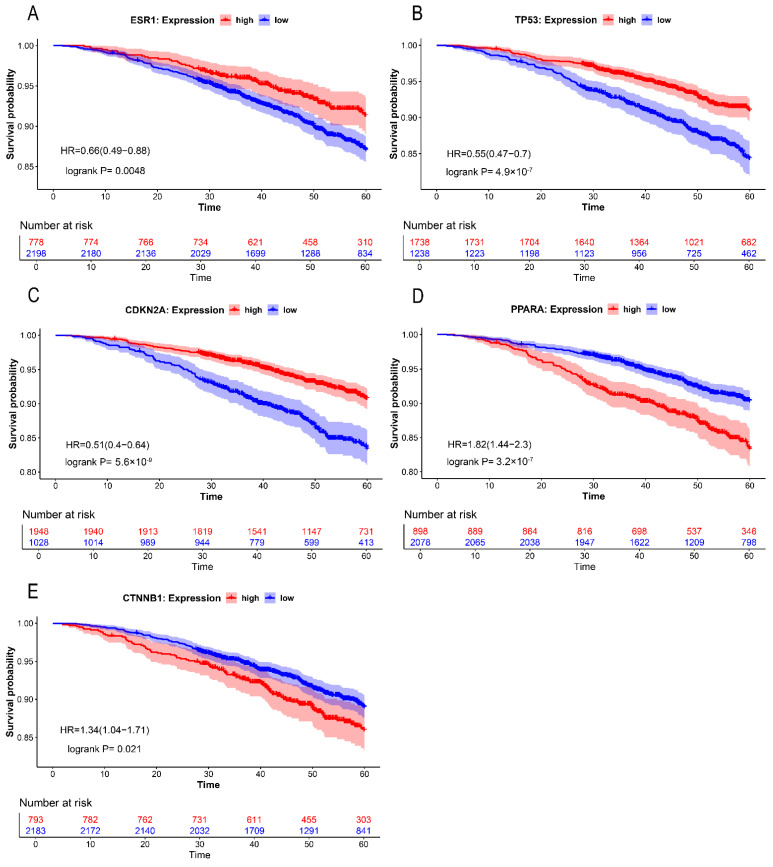
Kaplan–Meier survival curves stratified by hub gene expression. (**A**–**E**) correspond to *ESR1*, *TP53*, *CDKN2A*, *PPARA*, and *CTNNB1*, respectively. Survival probability (*y*-axis) is plotted against follow-up duration (months, *x*-axis). high-expression (red) and low-expression (blue) cohorts were defined by median transcript levels.

To assess the prognostic relevance of hub gene expression in breast cancer patients, Kaplan–Meier survival curves for overall survival (OS) were constructed and analyzed. The results showed that elevated expression of *ESR1*, *TP53*, and *CDKN2A* in BRCA patients was associated with improved OS (*p* = 0.0048, Figure 4A; *p* = 4.9 × 10^−7^, Figure 4B; *p* = 5.6 × 10^−9^, Figure 4C). Conversely, increased expression levels of *PPARA* and *CTNNB1* were significantly linked to worse OS (*p* = 3.2 × 10^−7^, Figure 4D; *p* = 0.021, Figure 4E). These findings demonstrated that the expression levels of these hub genes significantly influence the prognosis of BRCA patients (Figure 4).

### 2.5. Molecular Docking

**Figure 5:**
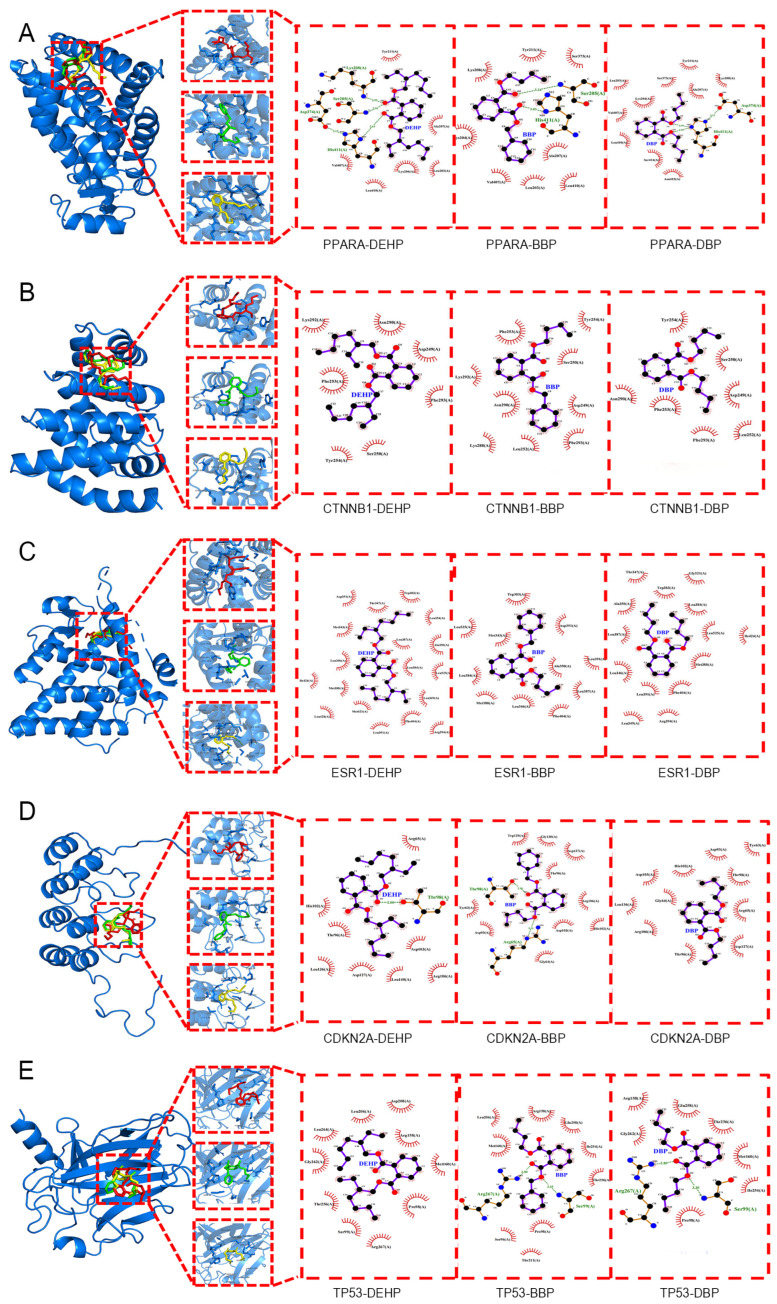
Molecular docking of hub genes with DBP (red), BBP (green), and DEHP (yellow). (**A**–**E**) correspond to *PPARA*, *CTNNB1*, *ESR1*, *CDKN2A*, and *TP53*, respectively.

Through AutoDock Vina, molecular docking was performed to assess the interactions between DBP, BBP, and DEHP with the five hub genes. The corresponding binding affinities are shown in Table 1. There is a strong direct binding interaction between the three types of PAEs and ESR1, all of which are less than −6.6 kcal/mol. These interactions between PAEs and the other four hub genes are also prevalent with the affinity no more than −4.4 kcal/mol, but BBP shows the strongest while DEHP shows the weakest one; the strongest binding associations were observed between PAEs and PPARA, while PAEs-CDKN2A shows the weakest one. Interactions primarily consist of extensive hydrophobic contacts and partial hydrogen bonding. As Figure 5 shows the most probable binding conformations between PAEs and hub genes.

As illustrated in Figure 5A–C, the binding sites of the three PAEs with oncogenes (PPARA, CTNNB1, and ESR1) are highly similar, but the hydrogen bonding is different. The interaction between PPARA and PAEs reveals widespread hydrogen bonding, shown in Figure 5A. Specifically, DEHP is observed to form hydrogen bonds with Lys208 (2.93 Å), Ser205 (2.19 Å), and His411 (2.93 Å). BBP exhibits a similar binding pattern while involving only Ser205 (3.24 Å) and His411 (3.04 Å). In contrast, DBP only forms two hydrogen bonds with His411 (2.99 Å). However, the interactions between PAEs and CTNNB1 or ESR1, shown in Figure 5B and Figure 5C, respectively, do not exhibit hydrogen bonding.

Figure 5D and Figure 5E illustrate the interactions between PAEs and tumor suppressor genes (CDKN2A and TP53), respectively. In the interaction with TP53, both BBP and DBP can form hydrogen bonds with Arg267 and Ser99, with the distances of 2.8 Å and 3.35 Å to BBP, respectively, and 2.8 Å and 3.3 Å to DBP, whereas DEHP does not exhibit such interactions. BBP forms hydrogen bonds with Arg65 and Thr98 of CDKN2A, with distances measured at 3.14 Å and 2.98 Å, respectively. DEHP forms hydrogen bonds with Thr98, with a distance of 2.88 Å. While no hydrogen bonds formed in the interaction between DBP and CDKN2A.

Overall, the interaction between PAE and ESR1 is the strongest stabilization, followed by PPARA. PAEs-ESR1 exhibited the lowest binding energy values, while PAEs-PPARA interactions were enhanced by extensive hydrogen bond networks. BBP demonstrated the highest binding ability with hub genes, while DEHP exhibited the lowest one.

### 2.6. Computer-Aided Molecular Design and Molecular Docking for Validation

**Figure 6:**
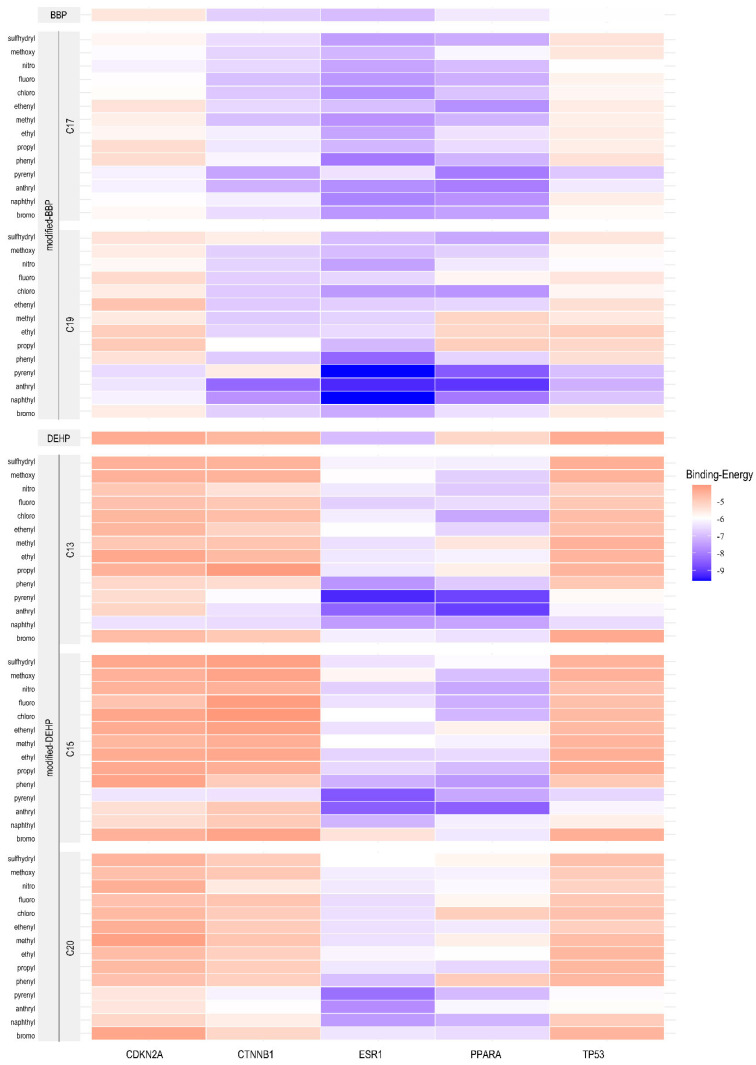
Heatmap of binding affinities for PAEs. Heatmap intensity reflects normalized binding affinities (−ΔG values) across evaluated targets, where darker blue signifies lower values (stronger binding) and darker red indicates higher values (weaker binding).

Given the observed differences in molecular docking capabilities between DEHP and BBP, we analyzed the functional groups of PAE responsible for the binding with the hub genes. PharmMapper-based pharmacophore identified two modification sites (C17 and C19) in BBP and three sites (C13, C15, and C20) in DEHP (C24 is no longer included due to structural symmetry with C15). Notably, the C19 site of BBP and the C13 site of DEHP are positioned at the terminal ends of their respective carbon chains. These sites were modified using 14 chemical groups to obtain new PAEs. The resulting modified PAEs were then evaluated for binding activity against proteins encoded by the hub gene identified in the network analysis. The interaction outcomes are visualized in Figure 6.

**Figure 7:**
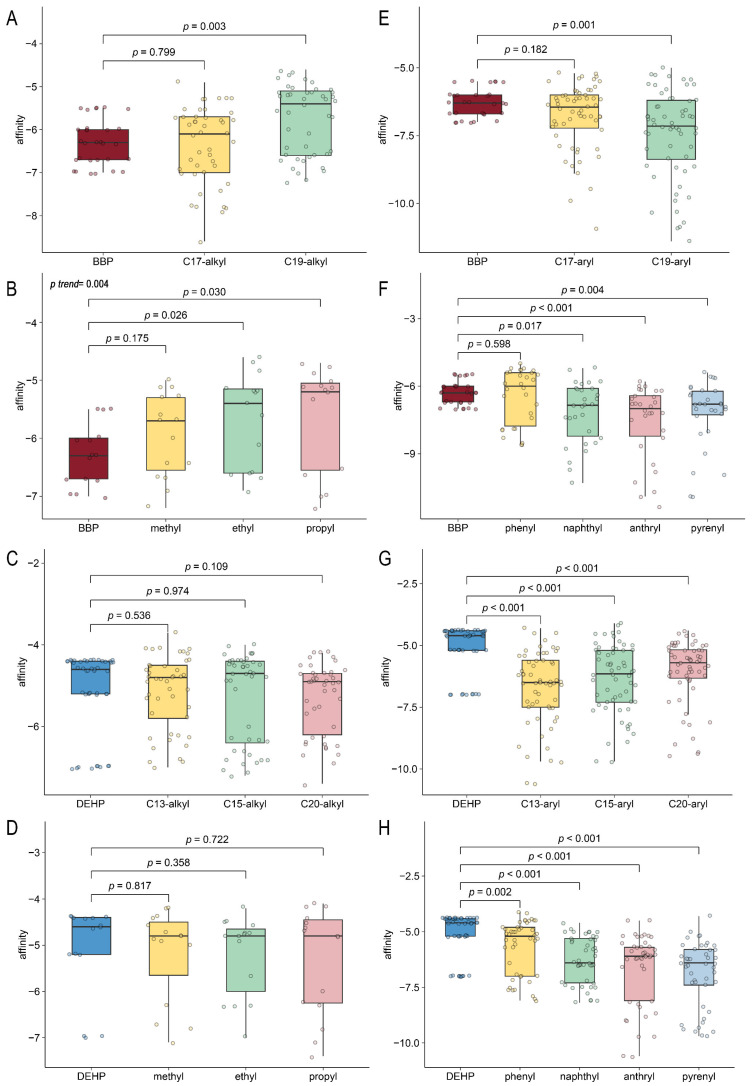
Affinity comparisons between modified PAEs and original PAEs. (**A**–**D**) Alkyl group modifications. (**E**–**H**) Aromatic substituent modifications. BBP derivatives (**A**,**B**,**E**,**F**), DEHP derivatives (**C**,**D**,**G**,**H**).

**Figure 8:**
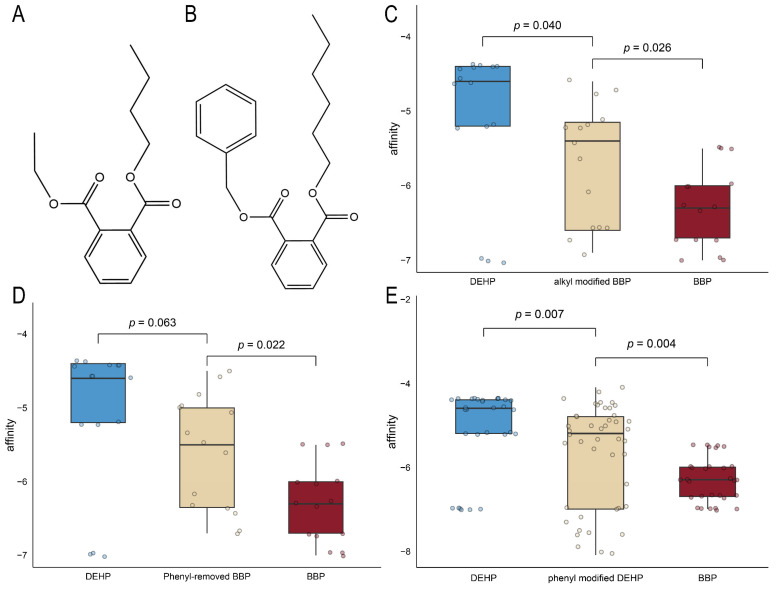
Affinity comparisons for modified PAEs with BBP and DEHP. Structural formula of modified BBPs: alkyl-modified BBP (**A**) and phenyl-removed BBP (**B**). Comparison for alkyl-modified BBP (**C**), comparison for phenyl-removed BBP (**D**), comparison for phenyl-modified DEHP (**E**).

The heatmap (Figure 6) illustrates binding energy through a color gradient, where darker blue signifies lower values (stronger binding) and darker red indicates higher values (weaker binding). For an alkyl group (methyl [–CH_3_], ethyl [–CH_2_CH_3_], propyl [–CH_2_CH_2_CH_3_]) modification, when it occurred at the C19 position of BBP, corresponding to side-chain elongation, the docking results exhibited darker red hues (Figure 6) with significantly increased binding energy (*p* = 0.003) (Figure 7A). The longer the alkyl chain extension in C19, the greater the binding energy values, corresponding to diminished binding affinity (Figure 7B, *p* = 0.004). In contrast, alkyl modifications at the C17 position of BBP (Figure 7A) and C13, C15, and C20 of DEHP (Figure 7C,D) show no comparable effect. On the other hand, aromatic substituents (phenyl [–C_6_H_5_], naphthyl [–C_10_H_7_], anthryl [–C_14_H_9_], and pyrenyl [–C_16_H_9_]) enhanced binding affinity for both BBP and DEHP, reflected as darker blue regions in the heatmap (Figure 6). In detail, only C19 aryl-modified variants significantly decreased the binding energy (Figure 7E), and non-phenyl aromatic substitutions in BBP exhibited notable effects (Figure 7F). While aromatic substitution at DEHP’s three positions (C13, C15, and C20) with four substituents (naphthyl, anthryl, pyrenyl, and phenyl) significantly reduced binding energy compared to the native structure (Figure 7G,H). These results indicate that side-chain elongation reduces binding affinity in short-chain PAE (BBP), whereas aromatic substitutions enhance affinity in longer-chain DEHP. To validate the hypothesis, alkyl-modified BBP (Figure 8A), phenyl-removed BBP (Figure 8B), and phenyl-modified DEHP were constructed and compared with native BBP and DEHP for binding affinity. Extending BBP’s carbon chain to match DEHP’s length (carbon-chain-modified BBP) significantly decreased binding affinity compared to native BBP (Figure 8C, *p* = 0.026), though it remained stronger than DEHP (*p* = 0.04). Removing BBP’s native benzyl group (phenyl-removed BBP) weakened binding affinity compared to native BBP (Figure 8D, *p* = 0.022), with no significant difference from DEHP (*p* = 0.063). Introducing phenyl groups at three sites on DEHP substantially enhanced its binding affinity compared to native DEHP (*p* = 0.007), though it was still lower than BBP (*p* = 0.004) (Figure 8E). These results indicate that the binding affinity disparity between BBP and DEHP arises from BBP’s shorter side chain and presence of a phenyl group.

## 3. Discussion

Phthalates (PAEs) are widely present in environmental compartments such as air, water, food, and personal care products, etc. [29], increasing the breast cancer risk of women exposed to PAEs [30] while obstructing effective prevention and control. This makes the study of common mechanisms urgent. Our study employed network toxicology to identify a PAE-promoting cancer regulatory model centered on hub genes associated with transcription factor activity (*ESR1*, *PPARA*, *CTNNB1*, *TP53*, *CDKN2A*). Molecular docking revealed that all three PAEs directly interacted with the hub gene, with different binding energies and hydrogen-bond counts. BBP showed the strongest direct binding, while DEHP showed the weakest. With the assistance of computer-aided molecular design and molecular docking, a group addition/subtraction controlled experiment revealed that the side chain length of DEHP and the phenyl group of BBP are the main functional groups that cause the difference in the binding force between them. These findings highlight the crucial role of transcription factor pathways in PAE-driven breast cancer promotion, identify PAE-hub gene interactions, and underscore the structure-activity relationship (SAR) to provide a theoretical basis for unified intervention such as target gene-based molecular intervention and structure-based novel PAE substitute development.

Network toxicology provides a systematic framework for elucidating the toxicological properties of chemical agents through the integration of enrichment analysis and network modeling. In fact, scholars laid out foundational methodologies and compiled relevant databases as early as 2012 [31] and then applied network analysis concepts to the field in 2014 [32]. Until recent years, network toxicology has developed a mature methodological framework [33], constructing regulatory networks that include identification of hub targets and critical signaling pathways, and has demonstrated progress in elucidating the mechanistic relationships between environmental toxicants and human health outcomes [34]. For instance, He et al. investigated the mechanisms underlying thiabendazole-induced cancer and liver injury, providing theoretical foundations for environmental policy formulation [35], and Zhang et al. assessed the impact of polyethylene terephthalate microplastics (PET-MPs) on intervertebral disc degeneration (IVDD), revealing common pathogenic mechanisms underlying microplastic-induced tissue damage [36]. Using network toxicology, this study explored the common targets underlying PAE-driven promotion of breast cancer progression. Functional enrichment analysis cued the transcription regulatory as crucial in mediating PAE effects. And KEGG pathway analysis indicated a marked enrichment for pathways related to cancer. Subsequently, through constructing the protein–protein interaction (PPI) network, we identified five hub genes, including *TP53*, *CTNNB1*, *PPARA*, *ESR1*, and *CDKN2A*. The results of differential expression and survival analyses provided robust support for five hub genes’ significant role in the progression and prognosis of breast cancer.

In fact, transcription factor regulation has been demonstrated to play an important role in the mechanisms underlying PAE-induced promotion of breast cancer and drug resistance [15,37]. Behaving as xenoestrogens, PAE can strongly bind to estrogen receptors to exert biological functions [38,39,40]. *PPARA* can be activated in MCF-7 cells by PAE [41]. *CTNNB1* expression was also found upregulated in ER+/HER2- breast cancer [42]. But *CTNNB1* was often reported to serve as a critical component of the Wnt/β-catenin signaling pathway to be implicated in various PAE-induced diseases [43,44]. Taken together, these three hub genes exert a substantial influence on breast cancer progression as oncogenes, which was supported by our study. Notably, in survival analysis, patients with high *ESR1* expression demonstrated significantly longer survival times compared to those with low *ESR1* expression. This phenomenon may be attributed to the fact that *ESR1*+ patients exhibit significant responsiveness to tamoxifen [45], and cancer women with *ESR1* amplification showed significantly prolonged survival following adjuvant tamoxifen monotherapy [46]. Among five hub genes, *TP53* and *CDKN2A* are well-known tumor suppressors [47]. However, mutations in *TP53* can abrogate its tumor-suppressive function. Even in the presence of wild-type *TP53*, overexpression of *MDM2* in breast cancer cells can bind to and inhibit *p53*, thereby promoting tumor progression [48]. Similarly, *CDKN2A* can lose its tumor-suppressive functions, also caused by mutations and other mechanisms [49].

Molecular docking was performed using AutoDock Vina 1.2.5, which calculates energy grids around a target site, a method widely used in biological research. A 2021 study employed SWISS-MODEL homology modeling techniques combined with molecular docking to explore the cellular mechanism of small-molecule enzyme targeting, exemplifying it as a reliable research approach [50]. According to the criterion that a binding affinity value less than −4.25 kcal/mol suggests the potential for ligand-receptor binding, our docking studies revealed successful ligand-receptor docking between PAEs and the protein products of hub genes, among which the bindings with ESR1 and PPARA were strongest (binding energy all below −5 kcal/mol). Previous studies have demonstrated that multiple PAEs (e.g., DEHP, BBP) exhibit direct binding interaction with ESR [40,51]. Buteau-Lozano et al. also reported that BBP and DEHP induce VEGF protein expression by an ER-dependent mechanism in breast cancer cells [52]. The direct molecular interactions between PAE and PPARA were also confirmed to exist in the occurrence of hepatocellular carcinoma in rodents [53].

In our study, all three PAEs showed a strong and stable binding with ESR1 and PPARA through strong binding force or extensive hydrogen bonding assistance, suggesting that these receptors may be primary targets through which breast cancer is promoted. However, quantitative analysis revealed that the three PAEs exhibited relatively weak binding affinity to CTNNB1, suggesting that the interaction may be transient and unlikely to form stable complexes in cellular environments. Therefore, indirect regulatory mechanisms likely represent the predominant pathway through which PAEs promote breast cancer progression via *CTNNB1* [43,44]. Although PAEs are capable of docking with the two tumor suppressor genes (*TP53*, *CDKN2A*), their binding affinity is comparatively weaker than that observed with *ESR1* and *PPARA*, likely due to the limited formation of hydrogen bonds except direct binding [54]. By binding to tumor suppressor gene products, PAEs may perturb hydrophobic and hydrophilic interactions, leading to conformational alterations that compromise the proteins’ tumor-suppressive functions. Furthermore, BBP demonstrated the highest binding, while DEHP exhibited the lowest one in our study. Further investigation is warranted to elucidate the reason underlying this differential binding affinity.

Computer-aided molecular design facilitates the identification of key modification sites (i.e., pharmacophores) through pharmacophore modeling [55]. In recent years, this methodology has evolved into a powerful strategy in toxicological mechanism research, with wide applications in both the elucidation of toxic mechanisms and the development of safer chemical alternatives [27,56]. A 2022 study has utilized pharmacophore to investigate the toxic mechanisms of PAEs and to design novel PAE analogs [26,56]. To elucidate the mechanisms underlying the significant differences in docking results between DEHP and BBP, we conducted molecular design based on pharmacophore theory. The results revealed that elongation of the carbon side chain at the C19 position of BBP led to a notable increase in binding energy; the aromatic substituent modification of DEHP can significantly decrease its binding energy, whereas other modifications of DEHP do not show significant changes. Therefore, it is presumed that the difference in binding affinity between BBP and DEHP was attributed to the difference in side chain length and the presence of an aromatic substituent. To verify this hypothesis, we constructed carbon-chain-modified BBP and phenyl-removed BBP to detect binding energy between modified PAE and hub genes. The results revealed that all modified BBPs exhibited significantly higher binding energies compared with the original BBP and showed no significant difference compared to DEHP. Concurrently, we found that increasing the side chain length of DEHP no longer reduces its interaction with hub genes, which suggests that the changes in binding activity caused by increased side chain length are limited. Research reports that the highest reproductive toxicity in PAEs is associated with side chains containing 5–6 carbons. A progressive decrease in this activity occurs with increasing chain length, culminating in its abolition at chain lengths of 8–13 carbons. Our conclusion confirms this structure-activity relationships (SARs) [57].

Since being identified as an endocrine-disrupting compound (EDC) [58], extensive research has been conducted to elucidate the relationship between PAE exposure and the development of breast cancer [59,60]. Building upon established network toxicology databases, our study highlights the key role of transcription factors in this process, identifies key hub genes associated with PAE-promoted breast cancer progression, and elucidates the molecular mechanisms underlying differential docking affinities of various PAEs. These findings provide a theoretical foundation for a deeper understanding of potential common mechanisms through which PAEs contribute to breast carcer and offer valuable insights for the rational design of targeted molecular modifications and the development of effective intervention strategies. The elucidation of structure-activity relationships between phthalate ester (PAE) molecular configurations and their oncogenic potential also provides critical proof for designing safer plasticizers.

There are also limitations to this study. Currently, the in vivo metabolites of PAEs were not investigated during molecular docking. Moreover, the number of PAEs we studied was limited, so caution should be exercised in extrapolating conclusions. Looking ahead, it is necessary to investigate whether there are significant differences in the roles of PAEs and their metabolites in the PAE-promoting cancer regulatory model and to expand the variety of PAEs studied. With the accumulation of research data and the updating of research methods, more generalizable conclusions will be drawn. Additionally, ADME predictions were not addressed in the present work, which will be prioritized in our future investigations.

## 4. Methods

The research strategy is illustrated in the flowchart shown as Figure S1.

### 4.1. Constructing Datasets and Identifying Interactive Genes

We employed the Comparative Toxicogenomics Database (CTD, http://ctdbase.org/, accessed on 15 February 2025) to identify potential genes associated with exposure to DBP, BBP, and DEHP. Queries were structured as follows: “Chemical = DBP and Organism = Homo sapiens,” “Chemical = BBP and Organism = Homo sapiens,” and “Chemical = DEHP and Organism = Homo sapiens.” The CTD database was then searched using the query “disease = breast cancer and organism = Homo sapiens,” which yielded a list of genes associated with breast cancer. Cytoscape 3.10.2 was used to visualize and analyze the PAE-target interaction networks, facilitating the identification of shared molecular pathways and gene targets associated with all three PAEs. Common genes between the DEHP, BBP, and DEP exposure groups and breast cancer were identified using Venny 2.1.0, thereby determining the common genetic targets [61,62].

### 4.2. Functional Enrichment Analysis (FEA)

Functional enrichment analysis (FEA) was conducted based on the Gene Ontology (GO) and Kyoto Encyclopedia of Genes and Genomes (KEGG) pathway databases, using Metascape (https://metascape.org, accessed on 15 February 2025) to identify significantly enriched elements [63]. The GO analysis encompassed biological processes (BP), molecular functions (MF), and cellular components (CC). The results were visualized through the website (http://www.bioinformatics.com.cn) [64].

### 4.3. Construction of Protein Interaction Network and Pathway Enrichment Analysis

A protein–protein interaction (PPI) network analysis was conducted to identify hub genes for further investigation. STRING (https://string-db.org/, accessed on 15 February 2025) was used to construct the PPI network, with a confidence score threshold set to 0.4 or higher [65] Cytoscape was employed to visualize the results. Employing betweenness centrality as a metric, the top five gene targets were identified and designated as hub genes (HG).

### 4.4. Protein Structure Retrieval and Homology Modeling

The 3D structures for *ESR1* (PDB ID: 7UJ8), *TP53* (PDB ID: 1UOL), *PPARA* (PDB ID: 5HYK), and *CTNNB1* (PDB ID: 7AFW) were retrieved from the RCSB Protein Data Bank (PDB; https://www.rcsb.org/, accessed on 23 February 2025). The selection was based on a predefined filter designed to optimize structural quality and biological relevance while minimizing induced-fit bias. Since the 3D structure of cyclin-dependent kinase inhibitor 2A (*CDKN2A*) has not been determined experimentally, homology modeling techniques were employed to construct its structure. Specifically, the FASTA sequence from the RCSB Protein Data Bank (PDB ID: 7OZT, chain B) was utilized as input in the SWISS-MODEL workspace (https://www.swissmodel.expasy.org/, accessed on 3 March 2025) [66] to construct the 3D structure of *CDKN2A* using the template with the highest target-template alignment score.

### 4.5. Differential Expression Analysis and Survival Analysis

The TCGA database was leveraged to analyze the expression differences of hub genes (*TP53*, *PPARA*, *CTNNB1*, *ESR1*, *CDKN2A*) between breast cancer tissues and adjacent non-cancerous tissues through the UALCAN portal (The University of Alabama at Birmingham Cancer Data Analysis Portals; https://ualcan.path.uab.edu/index.html, accessed on 7 January 2025), a robust tool based on TCGA datasets [67,68], and the statistical significance was determined using Welch’s *t*-test.

Survival analysis was performed using the KM Plotter platform (https://kmplot.com) to evaluate the impact of hub genes on overall survival [69]. KM plotter offers an extensive dataset of 2976 breast cancer patients, along with their corresponding overall survival profiles, in which patients were stratified into high and low expression groups based on median expression levels of the hub genes. The R programming language was employed for the visualization and statistical analysis of the data.

### 4.6. Molecular Docking

The chemical structures of DBP, BBP, and DEHP were retrieved from the PubChem database (https://pubchem.ncbi.nlm.nih.gov/, accessed on 22 March 2025). The 2D structures of the three PAEs are shown in Figure S2. The structures were imported into PyMOL 2.5.5 and AutoDockTools 1.5.7 for optimization, energy minimization, and hydrogen addition, after which the modified structures were prepared as ligand molecules. Typically, a binding affinity less than −4.25 kcal/mol signifies a potential ligand-receptor binding interaction, less than −5.00 kcal/mol signifies a strong interaction, and less than −7.00 kcal/mol signifies a robust interaction [70].

The structures of hub genes obtained from RCSB or Swiss-Model underwent preprocessing of protein chains and ligands, geometric structure optimization, hydrogen addition, and water molecule elimination to prepare the structures as receptors. For clarity, the gene names mentioned in molecular docking studies refer to the corresponding proteins expressed by the genes.

AutoDockTools 1.5.7 was utilized to define the docking box parameters. Docking simulations were subsequently conducted using AutoDock Vina [71], and the binding interactions were visualized through PyMOL 2.5.5 and LigPlot+ 2.2.8 to generate both 2D and 3D images of ligand–receptor complexes [72].

### 4.7. Computer-Aided Molecular Design

We employed a pharmacophore matching approach to identify pharmacophores for PAEs and subsequently modified their structures based on pharmacophore features. Molecular docking simulations were then conducted to identify critical functional groups influencing the binding interactions between PAEs and hub genes. Using the PharmMapper server (https://lilab-ecust.cn/pharmmapper/index.html, accessed on 12 April 2025) [73,74], PAEs were characterized by their pharmacophore features. To maintain the functional integrity of the PAEs as phthalates, we applied molecular modifications to the pharmacophoric sites without altering their core structures, which include the benzene rings and carboxylic acid groups. A total of fourteen types of functional groups were utilized to modify the PAE target molecules. These modifications included methyl (–CH_3_), ethyl (–CH_2_CH_3_), propyl (–CH_2_CH_2_CH_3_), phenyl (–C_6_H_5_), fluoro (–F), chloro (–Cl), bromo (–Br), nitro (–NO_2_), sulfhydryl (–SH), methoxy (–OCH_3_), ethenyl (–CH=CH_2_), naphthyl (–C_10_H_7_), anthryl (–C_14_H_9_), and pyrenyl (–C_16_H_9_) groups [26]. Molecular docking simulations were conducted between the modified PAEs and hub genes. All docking experiments included three independent runs, with subsequent statistical analysis and data visualization conducted using the R 4.4.1.

### 4.8. Statistical Analysis

Welch’s *t*-test was performed to analyze differences in the expression of hub genes between breast cancer tissues and normal tissues. The log-rank test was conducted to evaluate the association between hub gene expression and overall survival. The Wilcoxon test was used to analyze differences in binding affinities between modified and unmodified PAEs. *p*-values < 0.05 were considered statistically significant.

## 5. Conclusions

This study provides a comprehensive understanding of potential common mechanistic linkages between PAE (DBP, BBP, and DEHP) exposure and breast cancer through integrated bioinformatic analysis and molecular docking and explains the structural basis for the divergent molecular docking outcomes between BBP and DEHP via molecular design. These findings establish a theoretical foundation for our understanding of common mechanisms between environmental determinants and breast cancer and developing targeted unified intervention strategies.

## Figures and Tables

**Table 1:** Molecular docking affinity between PAEs and hub genes.

Affinity (kcal/mol)		Hub Genes				
		PPARA	CTNNB1	ESR1	CDKN2A	TP53
PAE	DEHP	−5.2	−4.6	−7	−4.4	−4.4
	DBP	−5.5	−5.3	−6.6	−5	−4.8
	BBP	−6.3	−6.7	−7	−5.5	−6

## Data Availability

Data will be made available on request.

## Supplementary Materials

The following supporting information can be downloaded at: https://www.mdpi.com/article/10.3390/ijms26209878/s1.
