# Supplementary material for: Computer-Aided Molecular Design Meets Network Toxicology and Molecular Docking: A Joint Strategy to Explore Common Molecular Mechanisms of Phthalates on Human Breast Cancer and Structure–Activity Relationship
Source: Int J Mol Sci. 2025 Oct 10;26(20):9878. doi: 10.3390/ijms26209878 (PMC12563439; doi:10.3390/ijms26209878)
Supplement: Supplementary file 1 [file ijms-26-09878-s001.zip › ijms-3906404-supplementary.pdf]

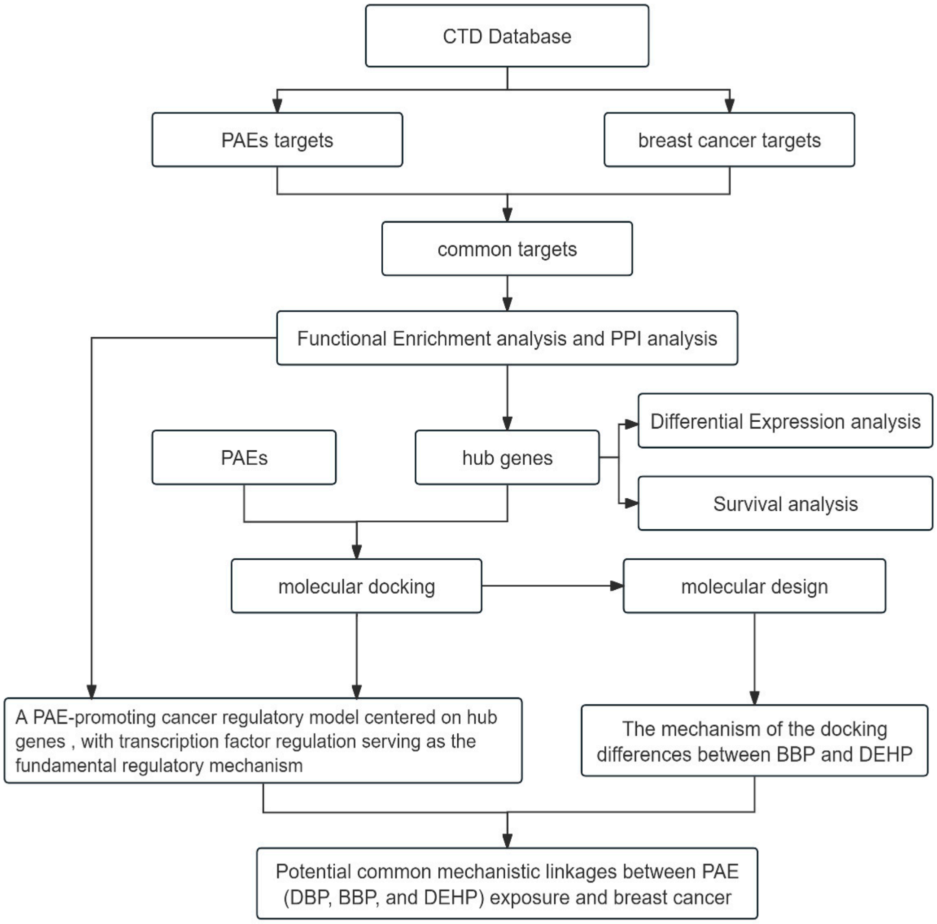

Figure S1. The flowchart of this study.

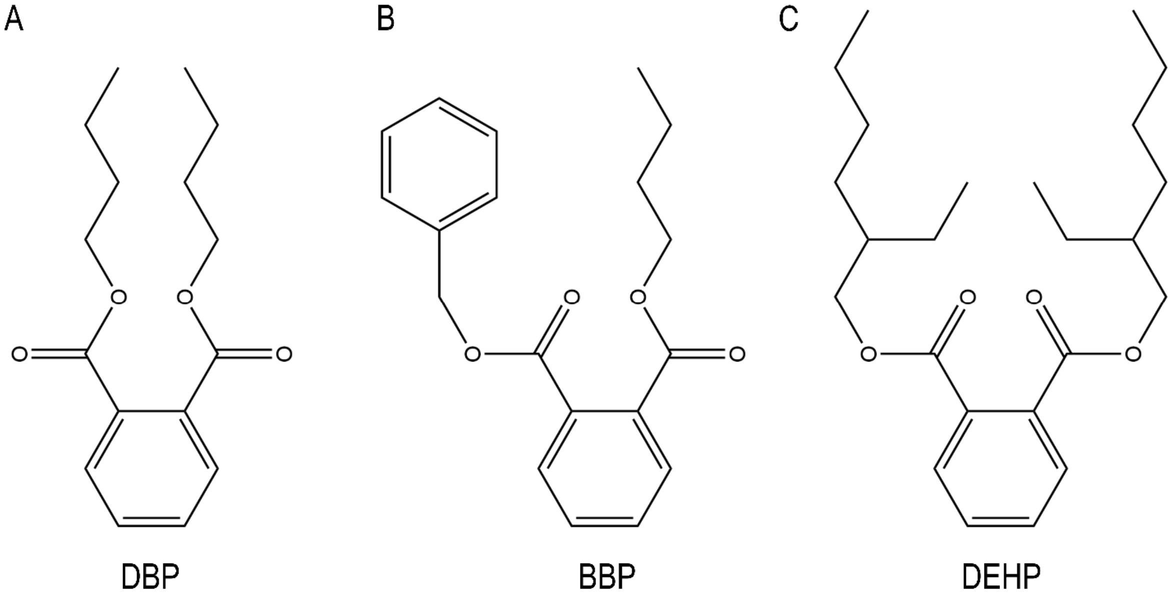

Figure S2. 2D chemical structures of the three PAEs employed in this study. (**A**) dibutyl phthalate (DBP), (**B**) benzyl butyl phthalate (BBP) and (**C**) di-(2-ethylhexyl) phthalate (DEHP).
